# The influence of patients’ nutritional risk, nutritional status, and energy density in MEDPass versus conventional administration of oral nutritional supplements – A secondary analysis of a randomized controlled trial

**DOI:** 10.1016/j.jnha.2024.100170

**Published:** 2024-02-02

**Authors:** Karin Schläppi, Emilie Reber, Katja A. Schönenberger, Zeno Stanga, Silvia Kurmann

**Affiliations:** a Department of Diabetes, Endocrinology, Clinical Nutrition and Metabolism, Inselspital, Bern University Hospital, University of Bern, Switzerland; b Division of Clinical Pharmacy and Epidemiology, Department of Pharmaceutical Sciences, University of Basel, Basel, Switzerland; c Health Division, Nutrition and Dietetics, Bern University of Applied Sciences, Bern, Switzerland

**Keywords:** MEDPass, Oral nutritional supplements, Nutritional risk screening 2002, Energy density, Energy coverage

## Abstract

**Objectives:**

The clinical influence of nutritional risk, nutritional status, and energy density of oral nutritional supplements (ONS) in MEDPass versus conventional administration of ONS is currently unknown. The aim of this analysis was to examine whether these variables have an impact on clinical outcomes.

**Methods:**

Secondary analysis of the intention to treat dataset of the randomized controlled MEDPass Trial in geriatric and medical inpatients. Patients in the intervention group received 4 × 50 ml ONS during the medication rounds (MEDPass mode), while those in the control group received ONS in a non-standardized manner. The examined endpoints included energy and protein coverage, ONS intake, handgrip strength (HGS), weight, appetite nausea and 30-day mortality. Three subgroup analyses for NRS 2002 total score (3, 4 or 5−7 points), NRS 2002 impaired nutritional status score (0, 1, 2 or 3 points) and energy density of the ONS (1.5 kcal/mL or 2 kcal/mL) were performed using linear and logistic regression with interaction and mixed effect models.

**Results:**

The data of 202 patients (103 women and 99 men) at nutritional risk (NRS total 2002 score ≥3), mean (SD) age 82.2 (6.5) years were included. There was no significant difference between the groups in the primary endpoint energy coverage in all three subgroup analyses. There were also no significant differences between the groups in the secondary endpoints of protein coverage, ONS intake, HGS, weight, appetite, nausea, and 30-day mortality.

**Conclusion:**

The MEDPass mode of ONS administration was not superior to the conventional mode of administration in this study. ONS with high energy density (≥2 kcal/mL) should be offered since current evidence shows a tendency towards improved appetite, increased ONS and increased energy intake.

## 1 Introduction

Disease related malnutrition (DRM) is a highly prevalent condition in geriatric and medical inpatients. At hospital admission, 20–60% of these patients are at risk of DRM [[1], [2], [3], [4], [5]]. DRM has negative impacts on clinical outcomes, such as body weight [6], handgrip strength (HGS) [7], length of hospital stay [8], morbidity [9] and mortality [6]. Hence, early screening for the risk of DRM at hospital admission and consecutive appropriate nutrition therapy are crucial [4,10,11]. The European Society of Clinical Nutrition and Metabolism (ESPEN) [12] recommends the use of the Nutritional Risk Screening 2002 (NRS 2002) [13] to assess the risk for DRM in adult inpatients. The NRS 2002 includes three parts: (i) nutritional status, based on weight loss, body mass index (BMI) and reduced general condition, and recent food intake, (ii) disease severity (stress metabolism due to the disease), and (iii) age. The first two parts are scored from 0 to 3 points with one additional point for age ≥70 years. The NRS 2002 total score ranges from 0 to 7 points. A score of ≥3 points indicates a risk for DRM. There is growing evidence that patients with higher NRS 2002 scores and with poor nutritional status may benefit better from nutrition therapy [11,[13], [14], [15], [16], [17], [18], [19]]. The effect of a nutrition therapy may be more profound in patients with lower BMI, weight loss prior to initiation of nutrition therapy, lower baseline energy intake and older age [20].

Oral nutritional supplements (ONS) are an efficacious intervention in nutrition therapy [9,21]. Recommendations from nutritional guidelines regarding timing and dosage of ONS are lacking and they are therefore usually administered in an unstandardized manner. Furthermore, compliance to ONS intake is often insufficient, leading to inadequate nutrition therapy [22,23]. Standardizing the administration mode of ONS may contribute to improved compliance [24]. In the Medication Pass Nutritional Supplement Program (MEDPass) administration mode, ONS are distributed in small volumes three to four times per day together with the medication rounds [24]. A systematic review reported preliminary positive results of MEDPass administration on compliance [24]. However, evidence regarding daily energy and protein intake, body weight and HGS is inconclusive while evidence regarding clinical outcomes such as appetite and nausea is lacking [24]. Further, the association between the MEDPass mode of ONS administration and the degree of nutritional risk and nutritional status has not yet been investigated. Energy density of ONS may also be relevant. In three studies, patients receiving energy-dense ONS administered in the MEDPass mode developed less nausea, improved appetite, body weight, HGS and energy and protein intake [[25], [26], [27]]. However, these findings are not sufficiently robust to derive recommendations for nutrition therapy. Therefore, we aimed to investigate the influence of the nutritional risk and nutritional status according to the NRS 2002 as well as the influence of energy density of ONS in the MEDPass versus conventional administration of ONS on relevant clinical outcomes in geriatric and medical inpatients.

## 2 Methods

This is a secondary analysis of the randomized controlled open-label MEDPass Trial [28]. The MEDPass Trial primarily investigated the difference in total energy intake compared to patients’ requirements in MEDPass versus conventional administration of ONS. Secondary outcomes included protein intake compared to patients’ requirements, volume of ONS intake, course of weight, HGS, appetite, nausea, length of hospital stay, and 30-day mortality. Patients included in the MEDPass Trial were at risk of malnutrition (NRS 2002 ≥ 3 points) and recruited from medical and geriatric wards of the Inselspital, Bern University Hospital from November 2018 to November 2021.

Patients in the MEDPass group received 50 ml of ONS four times per day with the medication rounds. Patients in the control group received ONS in the administration mode of conventional clinical practice between meals or after dinner distributed by gastronomic personnel. Randomization was stratified according to the NRS 2002 total score and the energy density of the ONS. Food and ONS intake were continuously assessed by gastronomic personnel responsible for clearing food and ONS. Further secondary outcomes were assessed on weekly study visits until discharge or for a maximum of 30 days, and 30-day mortality was followed up per phone call. The detailed methods and results of the trial are published elsewhere [28,29].

The present study investigated the following three subgroup analyses: NRS 2002 total score (3, 4 or 5−7 points), NRS 2002 impaired nutritional status score (0, 1, 2 or 3 points) and energy density of the ONS (1.5 kcal/mL or 2 kcal/mL). The primary endpoint was the mean energy coverage per day (kcal, % of calculated daily requirement) throughout the hospitalization. The secondary endpoints were the mean protein coverage per day (g, % of calculated daily requirement), the mean daily ONS intake (ml) the course of HGS (kg), body weight (kg), appetite and nausea (measured by visual analogue scale), and the 30-day mortality.

### 2.1 Data analysis

For the distribution of data, variables were visually assessed using QQ plots. Baseline characteristics were described for all subgroups, using number and percentages for categorical variables and mean (standard deviation) for continuous variables. Differences in baseline characteristics between the groups of each subgroup analysis were tested with chi-squared tests for categorical variables, *t*-tests for continuous variables with two subgroups, and single factor analyses of variance (ANOVA) for continuous variables with three or more subgroups. We used linear regression models with interactions to examine potential association between the different energy density of prescribed ONS, the different NRS 2002 total scores, the NRS 2002 subscores impaired nutritional status (X variables) on the mean energy and protein coverage as compared to the individual requirements throughout the hospitalisation and mean intake of ONS per day (Y variables). Mixed effect models were used to examine potential associations between the different in energy density of prescribed ONS, different NRS 2002 total scores and NRS 2002 subscores, impaired nutritional status (X variables) in the development of HGS, appetite, nausea, and weight (Y variables; visits were considered as a fixed effect and participants as a random effect). All models were adjusted for the stratification factors ONS density and NRS 2002 total score. To verify the requirements for the selected linear regression and mixed effects models, the residuals were examined with scatter- and QQ-plots. We reported adjusted differences of the coefficients, estimated means and corresponding 95% confidence intervals (Cl). Data analysis was carried out with the statistical program R (version 4.1.2) using the packages emmeans [30], see [31], car [32] and oddsratio [33].

## 3 Results

Of 204 patients enrolled in the MEDPass Trial, 202 were included in this secondary analysis. Two patients were excluded as one patient in the control group lacked ONS prescription and another patient in the MEDPass group lacked records of energy and protein intake. Table 1 shows the baseline characteristics of the study population, overall and according to subgroups analyses. The mean (SD) age was 82.2 (6.5) years, and 194 (96%) patients were admitted to the geriatric clinic. Hundred and three (51%) patients were female. Overall, 66 patients (33%) had an NRS 2002 total score of 3 points, 87 (43%) a score of 4 points and 49 (24%) a score of 5−7 points. In total, 109 (54%) received a 1.5 kcal ONS and 93 (46%) a 2 kcal ONS. There was no significant difference in age or disease category in any of the three subgroup analyses. In the subgroup analysis of NRS 2002 total score, participants with a lower score weighed significantly more (p = 0.009) and had significantly lower NRS 2002 impaired nutritional status scores (p < 0.01). In the subgroup analysis of NRS 2002 impaired nutritional status, the participants with a lower score weighed significantly more (p = 0.017) and had significantly lower NRS 2002 total scores (p < 0.01). Significantly more women had a higher NRS 2002 score for impaired nutritional status (p = 0.036).

**Table 1:** Baseline characteristics of the study population.

		NRS 2002 total score (points)				NRS 2002 impaired nutritional status (points)					ONS energy density (kcal/mL)	
Parameter	Overall	3	4	5−7		0	1	2	3		1.5	2
n (%)	202 (100)	66 (33)	87 (43)	49 (24)		13 (6)	96 (47)	74 (37)	19 (9)		109 (54)	93 (46)
Female, sex n (%) a	103 (51)	32 (48)	42 (48)	29 (59)		4 (31)	43 (45)	42 (57)	14 (74)	*	60 (55)	43 (46)
Age, years, mean ± SD b, c	82.2 ± 6.5	81.6 ± 6.7	82.7 ± 6.7	82 ± 5.8		78.8 ± 5.5	82.7 ± 6.5	82.4 ± 6.8	81.2 ± 5.7		82. 5 ± 6.6	81.9 ± 6.4
Geriatric ward, n (%) a	194 (96)	64 (98)	85 (98)	45 (92)		13 (100)	94 (98)	73 (99)	14 (74)	*	104 (95)	90 (97)
Disease category, n (%) a												
Gastrointestinal diseases	18 (9)	5 (8)	8 (9)	5 (10)		1 (8)	9 (9)	6 (8)	2 (10)		11 (10)	7 (7.5)
Infectious diseases	44 (22)	11 (17)	21 (24)	12 (24)		1 (8)	19 (20)	21 (28)	3 (16)		21 (19)	23 (25)
Cardiovascular diseases	44 (22)	11 (17)	23 (26)	10 (20)		3 (23.1)	24 (25)	15 (20)	2 (10)		28 (25)	16 (17)
Neurological diseases	14 (7)	3 (4)	6 (7)	5 (10)		1 (8)	7 (7)	5 (7)	1 (5)		8 (7)	6 (6)
Oncological diseases	13 (6)	3 (4)	5 (6)	5 (10)		2 (15)	5 (5)	4 (5)	2 (10)		8 (7)	5 (5)
Other diseases	69 (34)	33 (50)	24 (28)	12 (24)		5 (38)	32 (33)	23 (31)	9 (47)		33 (30)	36 (39)
BMI, kg/m^2^ mean, ± SD, b, c	24.4 ± 4.6	25.8 ± 5.1	23.7 ± 4.3	22.9 ± 4	*	27 ± 6.1	24.4 ± 4.4	23.9 ± 4.3	21.8 ± 4		24.5 ± 4.8	24.1 ± 4.5
NRS 2002 total score, points, n (%)^a^										**		
3						13 (100)	46 (47)	5 (7)	2 (10)		34 (31)	32 (34)
4						0	49 (50)	38 (51)	1 (5)		51 (47)	36 (39)
5−7						0	2 (2)	31 (42)	16 (84)		24 (22)	25 (27)
NRS 2002 impaired nutritional status, points, n (%) a					**							
0		13 (20)	0 (0)	0 (0)							7 (6)	6 (6)
1		46 (70)	48 (55)	2 (4)							55 (50)	41 (44)
2		5 (8)	38 (44)	31 (63)							38 (35)	36 (39)
3		2 (3)	1 (1)	16 (33)							9 (8)	10 (11)
Prescribed ONS density, n (%)^a^												
1.5 kcal/mL, n (%)	109	34 (51)	51 (59)	24 (49)		7 (54)	55 (57)	38 (51)	9 (47)			
2 kcal/mL, n (%)	93	32 (48)	36 (41)	25 (51)		6 (46)	41 (43)	36 (49)	10 (53)			

*p < 0.05, **p < 0.01.

a Chi-squared test.

b anova.

c t-test (for variables with only two groups).

### 3.1 NRS 2002 total score subgroup analysis

Energy coverage, protein coverage, and ONS intake were not significantly different between the NRS 2002 total score groups (Table 2). Neither was the course of HGS, body weight, appetite, and nausea (Table S3 supplementary material). In the group with a total score 5−7, the estimated mean energy coverage was 12.2% and the estimated mean protein coverage was 15.1% higher in the control group than in the MEDPass group, (Table 2). However, those differences were not significant. HGS tended to increase from visit to visit in the group with NRS 2002 total score 3 in the MEDPass and the control group. In the group with NRS 2002 total score 4, HGS did not change from visit to visit in any of the study arms. In the groups with NRS 2002 total score 5−7, HGS tended to decrease from baseline to the first visit and increased again by the second visit (Table S3 supplementary material). Body weight decreased in all three groups over the study period (Table S3 supplementary material). Patients in the MEDPass groups had insignificantly more appetite and less nausea than the control groups across all study visits (Table S3 supplementary material). The odds ratio for 30-day mortality could not be calculated in this subgroup analysis due to the small numbers of events (Table S4 supplementary material).

**Table 2:** Estimated means and 95% CIs of the linear regression models with interaction for the three subgroup analyses.

Subgroup analysis (points)	Parameter	Group	n	Energy coverage, %	Protein coverage, %	Intake ONS/d, ml
NRS 2002 total score (points)	3	MEDPass	32	81.4 (72.2−90.7)	99.9 (88.4−111)	169 (154−183)
		Control	34	78.6 (69.6−87.5)	95.6 (84.5−107)	184 (170−197)
	4	MEDPass	42	82.7 (74.6−90.8)	104 (93.9−114)	173 (159−184)
		Control	45	85.6 (77.9−93.4)	105 (95.3−115)	172 (159−183)
	5−7	MEDPass	25	81.1 (70.7−91.6)	98.5 (85.5−111)	171 (154−186))
		Control	24	93.3 (82.7−104)	113 (100.1−127)	164 (147−179)
NRS 2002 impaired nutritional status (points)	0	MEDPass	8	98.6 (79−118.2)	124 (99.7−148)	174 (143−204)
		Control	5	95.0 (70.9−119.2)	121 (90.7−151)	211 (173−248)
	1	MEDPass	45	85.9 (77.2−94.5)	106 (95.6−117)	168 (154−181)
		Control	51	87.0 (78.9−95)	106 (96.5−116)	170 (157−182)
	2	MEDPass	41	74.4 (65.8−83.1)	90.3 (79.6−101)	169 (156−182)
		Control	33	79.1 (69.8−88.5)	97.0 (85.4−109)	165 (151−180)
	3	MEDPass	5	85.8 (61.3−110.3)	113 (82.3−143)	177 (139−215)
		Control	14	91.8 (77−106.6)	107 (88.7−125)	186 (163−208)
ONS density (kcal/mL)	1.5	MEDPass	54	79.6 (72.4−86.8)	102 (92.7−111)	170 (159−181)
		Control	55	82.1 (74.9−89.3)	104 (94.7−113)	172 (161−183)
	2	MEDPass	45	84.5 (76.7−92.3)	101 (91.2−111)	169 (157−181)
		Control	48	88.7 (81.2−96.3)	104 (94.8−114)	173 (162−185)

### 3.2 Impaired nutritional status subgroup analysis

Energy and protein coverage as well as ONS intake were not significantly different between the NRS 2002 impaired nutritional status groups (Table 2). In the group with score 2, the estimated mean energy coverage in the control group was 4.7% higher than in the MEDPass group and in the group with score 3, it was 6% higher (Table 2). Both differences were not significant. Furthermore, the course of HGS, body weight, appetite, and nausea did not significantly differ between the four groups (Table S3 supplementary material). Body weight tended to decrease in all four groups over the study period (Table S3 supplementary material). Patients in the MEDPass groups had insignificantly more appetite and less nausea than the control group across all visits (Table S3 supplementary material). The odds ratio for 30-day mortality could not be calculated in this subgroup analysis due to the small numbers of events (Table S4 supplementary material).

### 3.3 ONS energy density subgroup analysis

Energy and protein coverage as well as ONS intake were not significantly different between the groups (Table 2). Neither were the course of HGS, body weight, appetite, and nausea (Table S3 supplementary material). Body weight decreased in both groups over the study period (Table S3 supplementary material). There was no significant difference regarding 30-day mortality (Table S4 supplementary material).

## 4 Discussion

In the MEDPass Trial [28], patients were almost exclusively recruited on the geriatric wards. Patients on the medical wards were hospitalised shorter and unscheduled. Their shorter length of stay disqualified some of them according to the eligibility criteria of hospitalisation for ≥3 days after nutritional screening. Furthermore, patients with unscheduled hospitalisations are often too unwell or too anxious to consent to participation in clinical trials [34]. Due to this selection bias, our results cannot be extrapolated to disciplines other than geriatrics.

We could not show any statistically significant differences in the investigated outcomes in any of the three subgroup analyses.

In the subgroup analysis for NRS 2002 total score, in the group with a total score of 5−7, the estimated mean energy coverage was 12.2% higher in the control group than in the MEDPass group. Similarly, patients with NRS total score of 5−7 reached 15.1% higher protein coverage in the control group. Although these differences were not significant, it may suggest that patients with NRS 2002 total score of 5−7 tend to benefit less from the MEDPass administration mode. Since there was only a slight difference of 7 ml in ONS intake in this group, it means that the MEDPass group showed a tendency to eat less than the control group.

There were also some interesting tendencies in the patients in the NRS 2002 impaired nutritional status subgroup analysis with score 2 and 3. In the group with NRS 2002 impaired nutritional status score 2, the estimated mean energy coverage in the control group was 4.7% higher than in the MEDPass group and in the group with NRS 2002 impaired nutritional status score of 3, the difference was 6% higher. Similarly, as in the group with NRS total score of 5−7, there was only a small difference of 4 ml and 9 ml in ONS intake suggesting that the MEDPass group tended to eat less than the control group. However, since the results were not significant, the influence on oral intake requires further investigation. Surprisingly, participants in the MEDPass group of the two groups described above (NRS 2002 total score 5−7 and NRS 2002 impaired nutritional status 2) had a little but not significantly more appetite and less nausea than patients in the control group. In the MEDPass Trial, appetite and nausea were collected once a week [28]. Therefore, it is possible that asking about nausea and appetite before and after each meal, as was done in the study by Rolls and Bell [35], may have provided a clearer picture of whether there is an association with ONS intake. Further, de Groot et al. [36] described that appetite and nausea in hospitalized elderly patients can also be influenced by factors such as disease comorbidities and medications. Consideration of these factors can be helpful in the early detection and prevention of DRM. Thus, including the severity of the disease and the medication of the participants into the statistical models of further investigations may be of particular interest. These parameters were not collected in the MEDPass Trial [29]. Another limitation in the MEDPass Trial study design poses the fact, that ONS in the MEDPass mode was distributed by nurses whereas gastronomic personnel distributed the ONS in the conventional mode. This might have skewed the results assuming more therapeutic messaging in the intervention group. In a focus group study unaffiliated with the MEDPass Trial, nurses stated, that motivational work regarding ONS intake is reduced with MEDPass administration [37]. Nonetheless, they assumed responsibility for patients taking their ONS independent of the administration mode [37].

To our knowledge, no randomized controlled trial has ever directly examined the influence of different NRS 2002 total scores, NRS 2002 impaired nutritional status scores or the influence of energy density on the investigated outcomes when ONS was administered in the MEDPass versus the conventional mode of administration. Therefore, it is difficult to compare previous studies with ours because most of them did not investigate on the MEDPass administration mode and the ones that did, all showed high risk of bias [24].

Nevertheless, some studies indicate that patients at higher risk of DRM benefit better from nutrition therapy. For example, in a secondary analysis of the «Effect of early nutritional support on Frailty, Functional Outcomes, and Recovery of malnourished medical inpatients Trial» (EFFORT), Tribolet et al. [38] reported a significant difference in protein coverage between NRS 2002 total score subgroups. Participants with higher NRS 2002 total score and with lower BMI achieved higher protein coverage. There was, however, no difference regarding energy coverage. Further, Hersberger et al. [39], showed an adjusted hazard ratio per point increase in the NRS 2002 total score of 1.22 regarding 30-day mortality. They also found, that patients with high nutritional risk (NRS 2002 total score of >4 points) showed the greatest benefit from nutrition therapy with respect to 30-day mortality [14]. This was not confirmed in our NRS 2002 total score subgroup analysis.

Evidence indicates that patients with lower nutritional status benefit more from nutrition therapy. For example, in the RCT of Bastow et al. [18] the patients with the lowest baseline body weight achieved significantly higher weight gain with enteral nutrition than those with higher baseline body weight. Further, the systematic review of Stratton [40] reported that the mean percentage weight gain and total energy intake of elderly nursing homes residents receiving ONS was greater in individuals with BMI < 20 kg/m^2^ than in individuals with a BMI > 20 kg/m^2^. Furthermore, Tribolet et al. [38] reported significantly higher protein coverage in patients with higher NRS 2002 impaired nutritional status score.

In contrast, in the RCT by Matheson et al. [41], in which hospitalized, malnourished older patients received daily 2 × 237 ml ONS between meals during hospitalization and up to 90 days after discharge, even mild to moderately malnourished patients achieved significant improvement in HGS.

Finally, the RCT of Potter et al. [17], in which participants in the intervention group received ONS in the MEDPass administration mode, the severely undernourished (BMI < 18.5 kg/m^2^) showed a statistically significant reduction in mortality in MEDPass versus conventional administration. Our results did not confirm a greater benefit from nutrition therapy in MEDPass versus conventional administration of ONS regarding patients’ nutritional status.

We were unable to confirm our assumption that patients that consumed a more energy-dense ONS would have a better appetite than those prescribed a less energy-dense ONS. Our assumption for better appetite with energy dense ONS is supported by the trial of Jukkola et al. [42] in which participants who received a 2 kcal/mL ONS in MEDPass mode or a 1.5 kcal/mL ONS between meals achieved significantly higher ONS intake in the MEDPass group. According to reassessment with the Mini Nutritional Assessment© compared to before the intervention, patients in the MEDPass group significantly improved their frequency of intake of protein containing foods and showed a trend towards a better score concerning the course of appetite. Also, in the before-after study by Hubbard et al. [43], patients first received ONS at 1.5−2 kcal/mL for three days and then ONS at 2.4 kcal/mL for a mean of four days. Total energy and protein intake (food and ONS) was significantly higher with the 2.4 kcal/mL ONS. In the randomized controlled study by Ter Wee et al. [44] elderly nursing home residents received a 2 kcal/mL ONS (125 ml per serving) in the intervention group and a 1.5 kcal/mL ONS (200 ml per serving) in the control group between meals for 9 weeks. A trend towards lower frequency nausea was observed in the energy dense, small volume group compared to the standard group. No statistically significant differences were found in energy and protein intake between the two groups.

Even though the evidence suggests advantages of higher density ONS, it could not be confirmed in our study.

The main results of the MEDPass Trial [28] showed that energy and protein coverage was high in the MEDPass as well as in the control group (energy: 82% and 85% respectively; protein: 101% and 104%, respectively) and therefore a potential effect regarding patients’ and study staff’s behaviour could be present due to the unblinded study design [[45], [46], [47]]. Since the same data were used in this study, the three subgroup analyses showed similar results. Mean energy and protein intakes were not significantly different in any of the three subgroup analyses, and ONS intakes were also quite high in all study arms.

Significant weight loss during hospitalization was observed in all study arms of the two NRS 2002 subgroup analyses and insignificant weight loss in the study arms of the ONS subgroup analysis despite a relatively short average hospital stay (7–9.8 days). This is consistent with other studies showing that hospitalization is nutritionally unfavourable for elderly patients even though the food offered in sufficient amounts to maintain energy balance [17,48]. In contrast, in the RCT of Schuetz et al. [4] in which the study duration was comparable to our study, patients’ weight increased. However, the patients in their study were on average about 10 years younger and older patients (≥70 years) appear to have an increased susceptibility to malnutrition, which may reflect age-related nutritional deficiencies possibly related to the decrease in lean body mass with age [13].

The circumstance that no differences were found for the endpoints weight and HGS in the three subgroup analyses in our study could possibly also be due to the short intervention duration [49]. Studies, in which nutrition therapy showed significant differences in weight and HGS were found lasted on average 6 weeks to 4 months [50,51] whereas in the MEDPass Trial ONS was prescribed for an average of 7–9.8 days [28]. On the other hand, the intervention duration is roughly comparable to the pilot study of Baumann et al. [25] which found a trend toward improvement in HGS after an intervention with 2 kcal/mL ONS in the MEDPass mode for 7.5 days. Patients also gained weight in the RCT by McWhirter and Pennington [52] in which they received either ONS or enteral nutrition for 7–11.8 days. However, regarding body weight, considerations must be given to the fact that the validity of weight measurements in clinical patients is highly susceptible to various factors [[53], [54], [55]].

For successful nutrition therapy, it is important to consider patient preferences [56]. In some studies, participants prioritized MEDPass over conventional mode of administration [57,58]. However, it is not clear from these studies findings whether this also applies to the subgroups studied here.

## 5 Conclusion

We were not able to show the superiority of MEDPass mode administration in geriatric inpatients at nutritional risk and did not find correlations between the NRS 2002 total score, the degree of impairment of the nutritional status or the energy density of ONS on the investigated clinical outcomes. In daily clinical practice, the choice of the ONS administration mode on geriatric wards should be guided by patient’s preferences to maximize compliance to nutrition therapy. ONS with high energy density (≥2 kcal/mL) should be offered since current evidence shows a tendency towards improved appetite and increased ONS as well as energy intake. Further well-powered investigations in geriatric and other populations are needed.

## Funding statement

This study was part of a master thesis and was not funded. The MEDPass Trial was supported by an investigator-initiated study grant from Abbott Laboratories, Columbus, OH, USA (grant number ANSW1701). Furthermore, the MEDPass Trial was funded by Inselspital University Hospital, Bern, Switzerland, Bern University of Applied Sciences, Health Department, Bern, Switzerland. The trial research team had full decision-making authority and responsibility concerning the entire investigator-initiated trial and the study course.

## Ethical standards

The Ethics Committee of the canton of Bern, Switzerland approved the protocol of the MEDPass Trial on October 15, 2018 under the number 2018-01512 (29). Informed consent was obtained from all study participants upon inclusion. Since the secondary analyses for this study evaluated the original data of the MEDPass Trial and the subgroup analyses performed were already provided in the study protocol and no additional data were collected, the ethical requirements were also met for this study.

## Disclosure statement

KS: KS has no competing interests to declare.

ER: has no competing interests to declare.

KAS: KAS has no competing interests to declare.

ZS: The institution of ZS received speaking honorariums and research support from Nestlé Health Science, Abbott Nutrition, Fresenius Kabi and B Braun.

SK: SK has received honorariums from Abbott Nutrition and Fresenius Kabi and consulting fees from Omanda Medical Nutrition.
