# Supplementary material for: The influence of patients’ nutritional risk, nutritional status, and energy density in MEDPass versus conventional administration of oral nutritional supplements – A secondary analysis of a randomized controlled trial
Source: J Nutr Health Aging. 2024 Feb 2;28(3):100170. doi: 10.1016/j.jnha.2024.100170 (PMC12880566; doi:10.1016/j.jnha.2024.100170)
Supplement: Supplementary file 1 [file mmc1.docx]

**Supplementary material**

Table S3: Estimated means and 95% CIs of the mixed effects models for the visit outcomes (baseline to week 2), for the subgroups

| Subgroup analysis | Parameter | Group | n | HGS | Weight | Appetite | Nausea |
| --- | --- | --- | --- | --- | --- | --- | --- |
| NRS 2002 total score  (points, week) | 3, baseline | MEDPass | 32 | 22.2 (19.6-24.8) | 75.2 (69.9-80.4) | 6.5 (5.8-7.2) | 8.8 (8.2-9.4) |
|  |  | Control | 34 | 21.7 (19.1-24.2) | 72.9 (67.5-78.2) | 6.2 (5.5-6.9) | 9.1 (8.5-9.7) |
|  | 3, week 1 | MEDPass | 26 | 22.4 (19.8-25) | 75 (69.8-80.3) | 6.4 (5.6-7.1) | 8.9 (8.3-9.6) |
|  |  | Control | 25 | 21.9 (19.3-24.4) | 72.7 (67.3-78.1) | 6.1 (5.3-6.8) | 9.2 (8.8-9.9) |
|  | 3, week 2 | MEDPass | 8 | 22.5 (19.6-25.4) | 73.9 (68.6-79.3) | 8.2 (6.6-9.6) | 9.4 (8.2-10.5) |
|  |  | Control | 2 | 22 (19.1-24.9) | 71.6 (66.2-77.1) | 7.9 (6.6-9.3) | 9.6 (8.4-10.8) |
|  | 4, baseline | MEDPass | 42 | 21 (18.6-23.4) | 67.7 (63-72.5 | 5.5 (4.9-6.2) | 8.9 (8.3-9.4) |
|  |  | Control | 45 | 20.5 (17.9-23.1) | 65.4 (60.8-70.1) | 5.2 (4.6-5.9) | 9.1 (8.6-9.7) |
|  | 4, week 1 | MEDPass | 31 | 21.2 (18.8-23.6) | 67.3 (62.6-72.2) | 5.9 (5.2-6.6) | 8.7 (8.2-9.3) |
|  |  | Control | 37 | 20.5 (17.9-23.1) | 65 (60.4-69.7) | 5.6 (5-6.3) | 9 (8.4-9.6) |
|  | 4, week 2 | MEDPass | 7 | 21 (18.3-23.7) | 66.4 (61.6-71.3) | 6.9 (5.6-8) | 9 (7.9-10) |
|  |  | Control | 6 | 20.5 (17.9-23.1) | 64.1 (59.4-68.9) | 6.5 (5.3-7.8) | 9.2 (8.1-10.3) |
|  | 5-7, baseline | MEDPass | 25 | 20.1 (17.2-23) | 64.2 (57.4-71) | 5.4 (4.7-6.2) | 8.5 (7.7-9.2) |
|  |  | Control | 24 | 19.6 (16.7-22.5) | 61.9 (54.9-68.8) | 5.2 (4.4-5.9) | 8.8 (8.1-9.5) |
|  | 5-7, week 1 | MEDPass | 20 | 20 (17-22.9) | 63.8 (57-70.6) | 5.7 (4.9-6.6) | 8.8 (8.1-9.6) |
|  |  | Control | 15 | 19.4 (16.5-23.3) | 61.5 (54.5-68.5) | 5.5 (4.6-6.3) | 9.1 (8.4-9.9) |
|  | 5-7, week 2 | MEDPass | 8 | 20.6 (17.4-23.8) | 63.2 (56.3-70.1) | 5 (3.6-6.4) | 8.7 (7.5-9.9) |
|  |  | Control | 2 | 20.1 (16.9-23.3) | 60.9 (53.8-67.9) | 4.7 (3.3-6.2) | 8.9 (7.7-10.2) |
| NRS 2002 impaired nutritional status  (points, week) | 0, baseline | MEDPass | 8 | 21 (15.5-26.5) | 76.1 (65.8-86.4) | 7.7 (6.2-9.1) | 9.9 (8.6-11.2) |
|  |  | Control | 5 | 20.5 (14.8-26.2) | 74.6 (63.8-85.4) | 7.5 (5.95-9) | 10.1 (8.8-11.4) |
|  | 0, week 1 | MEDPass | 7 | 20 (14.4-25.5) | 76.3 (66-86.5) | 7.3 (5.7-8.8) | 9.6 (8.2-10.9) |
|  |  | Control | 4 | 19.5 (13.8-25.2) | 74.7 (64-85.5) | 7.1 (5.5-8.7) | 9.8 (8.4-11.2) |
|  | 0, week 2 | MEDPass | 2 | 21.3 (15.1-27.6) | 75 (64.6-85.4) | 10.3 (7.1-13.4) | 10.9 (8.2-13.45) |
|  |  | Control | 0 |  |  |  |  |
|  | 1, baseline | MEDPass | 45 | 22.3 (19.7-24.9) | 69.4 (64.1-74.2) | 6.23 (5.58-8.9) | 9.2 (8.7-9.8) |
|  |  | Control | 51 | 21.8 (19.3-24.4) | 67.9 (62-73.8) | 6.04 (5.38-6.7) | 9.4 (8.9-10) |
|  | 1, week 1 | MEDPass | 35 | 22.3 (19.8-24,9) | 68.9 (63.6-74.2) | 6.7 (5.98-7.4) | 9.3 (8.7-9.9) |
|  |  | Control | 38 | 21.9 (19.3-24.5) | 67.4 (61.5-73.3) | 6.5 (5.8-7.2) | 9.5 (8.9-10.1) |
|  | 1, week 2 | MEDPass | 7 | 21.9 (19-24.8) | 67.3 (61.9-72.6) | 6.9 (5.4-8.3) | 8.9 (7.8-10.2) |
|  |  | Control | 3 | 21.4 (18.5-24.4) | 65.8 (59.8-71.8) | 6.7 (5.2-8.1) | 9.2 (8-10.4) |
|  | 2, baseline | MEDPass | 41 | 20.4 (17.8-23) | 67.7 (62.2-73.3) | 5.15 (4.49-5.8) | 8.2 (7.6-8.7) |
|  |  | Control | 33 | 20 (17.3-22.7) | 66.4 (60.5-72.4) | 4.96 (4.28-5.7) | 8.3 (7.8-9) |
|  | 2, week 1 | MEDPass | 32 | 20.6 (18-23.3) | 67.7 (61.9-73.4) | 5.2 (4.5-5.9) | 8 (7.4-8.6) |
|  |  | Control | 25 | 20.2 (17.5-22.9) | 66.2 (60.2-72.1) | 5 (4.3-5.7) | 8.2 (7.6-8.8) |
|  | 2, week 2 | MEDPass | 14 | 20.9 (18.2-23.7) | 67.2 (61.4-73) | 6.1 (5-7.1) | 8.6 (7.7-9.5) |
|  |  | Control | 5 | 20.5 (17.6-23.3) | 65.7 (59.7-71.6) | 5.9 (4.8-7) | 8.8 (7.9-9.7) |
|  | 3, baseline | MEDPass | 5 | 18.9 (13.9-23.9) | 63 (51.4-74.6) | 5.05 (3.74-6.4) | 8.5 (7.4-9.6) |
|  |  | Control | 14 | 18.4 (13.9-23) | 61.5 (51.1-71.9) | 4.86 (3.64-6.1) | 8.7 (7.7-9.8) |
|  | 3, week 1 | MEDPass | 3 | 19.8 (14.7-24.8) | 62.7 (51-74.3) | 5 (3.53-6.47) | 9.8 (8.6-11.1) |
|  |  | Control | 10 | 19.3 (14.7-23.9) | 61.2 (50.7-71.6) | 4.8 (3.4-6.2) | 10 (8.8-11.2) |
|  | 3, week 2 | MEDPass | 0 |  |  |  |  |
|  |  | Control | 2 | 19.6 (14.1-25.1) | 60.8 (50-71.6) | 4.9 (1.8-8) | 9.5 (6.9-12.1) |
| ONS density  (kcal/ml, week) | 1.5, baseline | MEDPass | 54 | 20.9 (18.7-23.2) | 67.7 (62.9-72.4) | 5.6 (5.1-6.2) | 8.8 (8.3-9.3) |
|  |  | Control | 55 | 20.4 (18.2-22.6) | 65.4 (60.6-70.1) | 5.4 (4.8-5.9) | 9 (8.5-9.5) |
|  | 1.5, week 1 | MEDPass | 40 | 20.8 (18.5-23) | 67.2 (62.5-72) | 5.7 (5.1-6.3) | 8.7 (8.2-9.3) |
|  |  | Control | 42 | 20.3 (18.1-22.4) | 64.9 (60.2-69.6) | 5.4 (4.8-6.1) | 9 (8.4-9.5) |
|  | 1.5, week 2 | MEDPass | 14 | 20.7 (18.3-23.1) | 66.5 (61.7-71.3) | 6.3 (5.2-7.3) | 9 (8.1-9.9) |
|  |  | Control | 4 | 20.1 (17.8-22.5) | 64.2 (59.4-68.9) | 6 (4.9-7.1) | 9.2 (8.3-10.2) |
|  | 2, baseline | MEDPass | 45 | 21.3 (19-23.5) | 70.4 (65.7-75.1) | 6 (5.4-6.6) | 8.7 (8.2-9.2) |
|  |  | Control | 48 | 29.7 (18.5-23) | 68.1 (63.2-73) | 5.7 (5.1-6.3) | 9 (8.5-9.5) |
|  | 2, week 1 | MEDPass | 37 | 21.6 (19.4-24.2) | 70.2 (65.6-74.9) | 6.3 (5.6-7) | 8.9 (8.4-10.3) |
|  |  | Control | 35 | 21.1 (18.8-32.4) | 67.9 (63-72.8) | 6 (5.4-6.7) | 9.2 (8.6-9.8) |
|  | 2, week 2 | MEDPass | 9 | 22.2 (19.6-24.7) | 69.1 (64.4-73.9) | 7.1 (5.9-8.2) | 9.1 (8-10) |
|  |  | Control | 6 | 21.6 (19.1-24.2) | 66.8 (61.8-71.8) | 6.8 (5.6-8) | 9.3 (8.3-10.3) |

| Subgroup analysis | Parameter | Group | n | 30-day mortality, n (%) |
| --- | --- | --- | --- | --- |
| NRS 2002 total score  (points) | 3 | MEDPass | 32 | 0(0) |
|  |  | Control | 34 | 1 (2) |
|  | 4 | MEDPass | 42 | 0 (0) |
|  |  | Control | 45 | 5 (6) |
|  | 5-7 | MEDPass | 25 | 3 (6) |
|  |  | Control | 24 | 2 (4) |
| NRS 2002 impaired nutritional status  (points) | 0 | MEDPass | 8 | 0 (0) |
|  |  | Control | 5 | 1 (8) |
|  | 1 | MEDPass | 45 | 0 (0) |
|  |  | Control | 51 | 2 (2) |
|  | 2 | MEDPass | 41 | 2 (3) |
|  |  | Control | 33 | 5 (7) |
|  | 3 | MEDPass | 5 | 1 (5) |
|  |  | Control | 14 | 0 (0) |
| ONS density  (kcal/ml) | 1.5 | MEDPass | 54 | 1 (1) |
|  |  | Control | 55 | 5 (5) |
|  | 2 | MEDPass | 45 | 2 (2) |
|  |  | Control | 48 | 3 (3) |

**Table S4: 30-days mortality**
